# New hardware and workflows for semi-automated correlative cryo-fluorescence and cryo-electron microscopy/tomography

**DOI:** 10.1016/j.jsb.2016.06.020

**Published:** 2017-02

**Authors:** Martin Schorb, Leander Gaechter, Ori Avinoam, Frank Sieckmann, Mairi Clarke, Cecilia Bebeacua, Yury S. Bykov, Andreas F.-P. Sonnen, Reinhard Lihl, John A.G. Briggs

**Affiliations:** a Structural and Computational Biology Unit, European Molecular Biology Laboratory, Meyerhofstrasse 1, 69117 Heidelberg, Germany; b Electron Microscopy Core Facility, European Molecular Biology Laboratory, Meyerhofstrasse 1, 69117 Heidelberg, Germany; c Leica Microsystems (Schweiz) AG, Max Schmidheiny-Strasse 201, 9435 Heerbrugg, Switzerland; d Leica Microsystems GmbH, Am Friedensplatz 3, 68165 Mannheim, Germany; e Cell Biology and Biophysics Unit, European Molecular Biology Laboratory, Meyerhofstrasse 1, 69117 Heidelberg, Germany; f Molecular Medicine Partnership Unit, EMBL/Universitätsklinikum Heidelberg, Heidelberg, Germany; g Leica Mikrosysteme GmbH, Hernalser Hauptstraße 219, 1170 Vienna, Austria

**Keywords:** CLEM, correlative light and electron microscopy, CEMOVIS, cryo-electron microscopy of vitrified sections, EM, electron microscopy, ET, electron tomography, FM, fluorescence microscopy, FOV, field of view, LN, liquid nitrogen, NA, numerical aperture, POI, position(s) of interest, TEM, transmission electron microscopy, WD, working distance, Correlative light and electron microscopy, Cryo-fluorescence microscopy, Cryo-electron microscopy, Cryo-electron tomography

## Abstract

Correlative light and electron microscopy allows features of interest defined by fluorescence signals to be located in an electron micrograph of the same sample. Rare dynamic events or specific objects can be identified, targeted and imaged by electron microscopy or tomography. To combine it with structural studies using cryo-electron microscopy or tomography, fluorescence microscopy must be performed while maintaining the specimen vitrified at liquid-nitrogen temperatures and in a dry environment during imaging and transfer. Here we present instrumentation, software and an experimental workflow that improves the ease of use, throughput and performance of correlated cryo-fluorescence and cryo-electron microscopy. The new cryo-stage incorporates a specially modified high-numerical aperture objective lens and provides a stable and clean imaging environment. It is combined with a transfer shuttle for contamination-free loading of the specimen. Optimized microscope control software allows automated acquisition of the entire specimen area by cryo-fluorescence microscopy. The software also facilitates direct transfer of the fluorescence image and associated coordinates to the cryo-electron microscope for subsequent fluorescence-guided automated imaging. Here we describe these technological developments and present a detailed workflow, which we applied for automated cryo-electron microscopy and tomography of various specimens.

## 1 Introduction

Correlative light and electron microscopy (CLEM) combines the advantages of fluorescence microscopy (FM) and electron microscopy (EM). FM provides positional as well as dynamic information on specific biomolecules. EM provides detailed, high-resolution information on cellular ultrastructure and protein structure, while also revealing the environment surrounding the molecule of interest. This makes EM and its extensions to 3D volume imaging a powerful tool for structural biology as well as the method of choice for detailed analysis of cellular morphology (Carroni and Saibil, 2016, Lučič et al., 2013).

A biological sample needs to undergo preparatory steps before it can be imaged by EM. The goal is to maintain the sample in a close-to-native state and ensure optimal structure preservation while enabling it to enter the high vacuum microscope column. In “traditional” EM the sample is dehydrated and embedded in resin prior to staining with heavy metals and imaging at room temperature. In contrast, in cryo-EM the sample is imaged in its fully hydrated, vitrified state. The samples can be prepared by quickly plunging them into ultra-cold liquefied gases (Dubochet et al., 1988, Taylor and Glaeser, 1974), or by freezing them under high-pressure (McDonald, 2009). For samples that are too thick for cryo-EM imaging (above ∼500 nm), thin slabs can be cut from the sample by cryo-sectioning (Al-Amoudi et al., 2004) or the sample can be milled with a focussed ion beam (Rigort et al., 2012). Contrast is generated directly by the electron density differences within the molecules. Hence, cryo-EM permits the morphological and structural characterisation of biological samples near to their native state and at high-resolution (Carroni and Saibil, 2016). In electron tomography (ET), 2D projection images acquired at different specimen angles are combined to generate a 3D reconstruction of the imaged volume (Irobalieva et al., 2016, Lučič et al., 2013).

In recent years various CLEM techniques using traditional sample preparation methods have provided remarkable insights into different areas of cell biology (Briggs and Lakadamyali, 2012, Bykov et al., 2016, Gibson et al., 2014, Redemann and Müller-Reichert, 2013, Sjollema et al., 2012). To maximize the accuracy with which fluorescence signals are identified in an EM image, both FM and EM need to be performed on the identical specimen (Kukulski et al., 2012, 2011). This raises a particular challenge in correlating FM with cryo-EM: the FM imaging needs to happen at temperatures below −140 °C where the cryo-EM sample does not devitrify. In addition, the sample must be shielded from atmospheric humidity during imaging and loading, while mechanical motion and optical distortions due to cooling or temperature gradients must be minimized. A number of solutions have been developed that add a special cryo-stage to a standard or adapted fluorescence microscope for cryo-CLEM (Zhang, 2013). Currently available solutions that fit an inverted fluorescence microscope feature a glass slide that separates the cold sample from the microscope objective lens (Sartori et al., 2007, Schellenberger et al., 2014). The minimum working distance (WD) reported for an inverted cryo-stage system is 410 μm with a 0.9 numerical aperture (NA) objective lens (Arnold et al., 2016). Solutions that attach to an upright microscope geometry do not require a separating glass slide (Liu et al., 2015, van Driel et al., 2009). The optical performance of all of these systems is limited by use of an immersion-free objective lens with relatively high WD and correspondingly low NA (Briegel et al., 2010, Koning et al., 2014). In a previous study using an upright geometry, we have shown that a short-WD dry objective lens with a high NA can be used for high-accuracy cryo-CLEM with a specially designed stage in which the objective approaches the sample closely (Schorb and Briggs, 2014).

We have developed a new dedicated cryo-FM system for CLEM. Our main goals during development of this system were: 1) to improve the transfer of the sample to and from the cryo-stage to reduce contamination by atmospheric moisture and to avoid sample loss due to warming or otherwise failed transfers; 2) to provide a mechanically and thermally stable stage appropriate for imaging multiple samples over long periods of time; 3) to maximize the optical performance; and 4) to incorporate the new hardware and its control software into a workflow that allows automated FM scans of entire cryo-EM grids and easy transfer of FM images and coordinates to the EM for use during data collection. Here, we describe and assess the new cryo-FM system. We provide a detailed workflow and protocol for using the cryo-FM system to automatically image an entire EM grid; for transfer of coordinates from FM to the software controlling the electron microscope; for cryo-EM or cryo-ET data acquisition; and for the image registration steps required to maximize the accuracy of correlation.

## 2 Results

### 2.1 Design of the stage and transfer shuttle

**Fig. 1:**
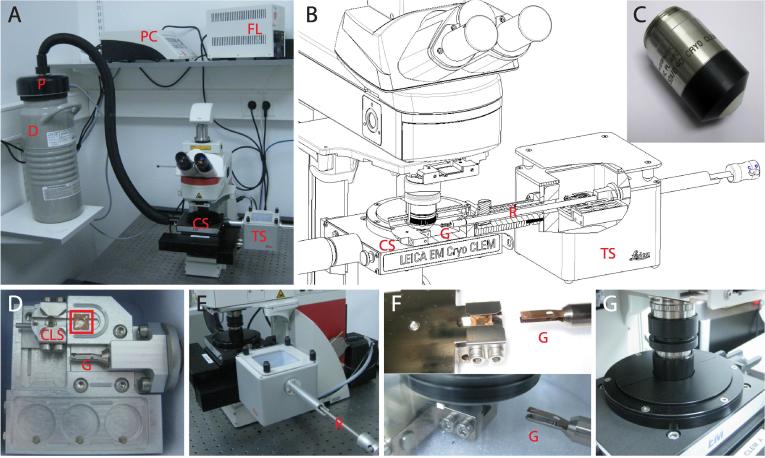
The cryo-FM stage and transfer shuttle. **A** Photograph of the microscope, cryo-stage (CS) and the mounted transfer shuttle (TS), the LN-pump (P) in a 5L dewar (D), the pump controller (PC) and the fluorescence light source (FL). **B** Cutaway drawing of the cryo-stage (CS) and mounted transfer shuttle (TS). The rod (R) with the gripper (G) is extending through airlocks into the stage. **C** Photograph of the modified cryo-microscopy objective lens. The black and white ceramic cover towards the front of the lens drastically reduces thermal conductance. **D** Detail of the transfer shuttle. The cartridge that holds the EM grid is marked with a red box. The cartridge loading station (CLS) is shown immediately left of it; the gripper (G) that inserts the cartridge into the cryo-stage can be seen in the center. The circular slots at the bottom hold grid storage boxes. **E** Side view of the transfer shuttle attached to the cryo-stage during the loading/unloading of a specimen. **F** Upper panel shows a close-up view of the specimen inside the cryo-stage. The gripper (G) reaches inside the stage to place or pick up the cartridge from the cooled support shown on the left. Lower panel shows stage with lid on and objective inserted. **G** Detailed view of the cryo-stage with inserted objective. The black collar around the objective prevents condensation from entering the stage through the objective tube. The transparent lid is closed with covers to prevent stray light from entering the chamber.

The cryo-FM stage is designed such that it can be mounted on a commercial upright microscope body (DM6FS, Leica Microsystems, Wetzlar, Germany). In this configuration the stage is fixed in *Z* but can move in *X* and *Y* directions, while the objective is inserted into the stage from above and moves axially for focusing. An overview of the stage and transfer shuttle design is given in Fig.1. Inside the stage, liquid nitrogen (LN), provided by an external pump, cools a metal block that supports the specimen. The interior of the stage is thermally isolated from the stage casing by the use of non-conductive materials. A high NA (0.9), short WD objective enters the stage through a port in the stage lid that tightly surrounds the objective (Fig.1G). The objective and the enclosing lid move relative to the flat stage surface during lateral (*X* and *Y*) movements while maintaining a seal to prevent influx of air. The front part of the objective is made of ceramic material with little thermal conductance to minimize heat transfer to the sample during imaging (Fig.1C). To avoid condensation inside the objective from environmental humidity, the spring mechanism for the front lens assembly was removed and the whole objective lens was sealed. In addition, a glass slide was added in the parfocal adapter that connects the objective lens and the microscope body to avoid build-up of condensation on the objective back lens. There is no intermediate cover glass between the objective and the sample, allowing the front lens to approach to 280 μm from the sample.

The sample is loaded into the stage using a transfer shuttle that can be mounted onto the side of the stage (Fig.1A, B, E). The transfer shuttle is filled with LN that cools the loading assemblies and provides a cold, dry environment to transfer samples from storage containers to the cryo-FM stage and vice versa. A transparent lid with spacers covers the transfer station when not in active use.

Inside the transfer shuttle, the vitrified samples on cryo-EM grids are loaded into a specialized, commercial, flat, square, copper cartridge for cryo-FM imaging (Leica 16707511109) (Movie S1). The transfer shuttle contains three spaces for standard cryo-EM grid storage boxes (Fig.1D). A grid can be removed from a storage box and mounted into the cryo-FM cartridge using a dedicated loading station. In the loading station thin copper clips that secure the grid inside the cartridge (Fig.1D) are raised, allowing the grid to slide into its position within the cartridge, and lowered to secure it in place. These copper clips have minimized material thickness in order to allow the objective to approach the sample as closely as possible. (Other available cartridge systems for cryo-EM secure the grid using a clip ring assembly whose thickness prevents FM imaging at low WD.) This configuration provides mechanical stability and protection during loading and handling of the sample. The grid stays mounted in the cartridge during transfer and imaging. The cartridge loading station is detachable and can be baked-out separately from the transfer shuttle.

The transfer shuttle incorporates an extendable rod with a gripper at its tip into which the cartridge can be inserted. To transfer the sample into the cryo-FM, the shuttle is docked to the stage, the rod is extended through a port in the wall of the shuttle directly into the cryo-FM stage and the gripper is released, leaving the cartridge in the stage (Fig.1F). The transfer shuttle is removed before imaging. During imaging the cartridge sits on top of an LN-cooled copper block. The design is such that vibrations from the pump or the evaporation of LN are minimized. A temperature sensor regulates the pumping rate and LN flow. A heater element is embedded in the copper block for adjusting the temperature and for baking out the system after use. A hole in the block underneath the specimen allows brightfield imaging. The vaporising dry nitrogen gas creates an overpressure atmosphere in the stage and prevents contamination by frost from condensing atmospheric humidity.

The microscope is controlled using a dedicated CLEM module that we integrated into Leica’s existing MatrixScreener HCSA (High Content Screening Automatization) software. This module provides the required functionality for generating grid-wide scans of cryo-EM specimens. The generated data format allows a direct exchange of images and coordinate lists with the software controlling the electron microscope.

### 2.2 A workflow for correlative cryo-FM and cryo-EM

**Fig. 2:**
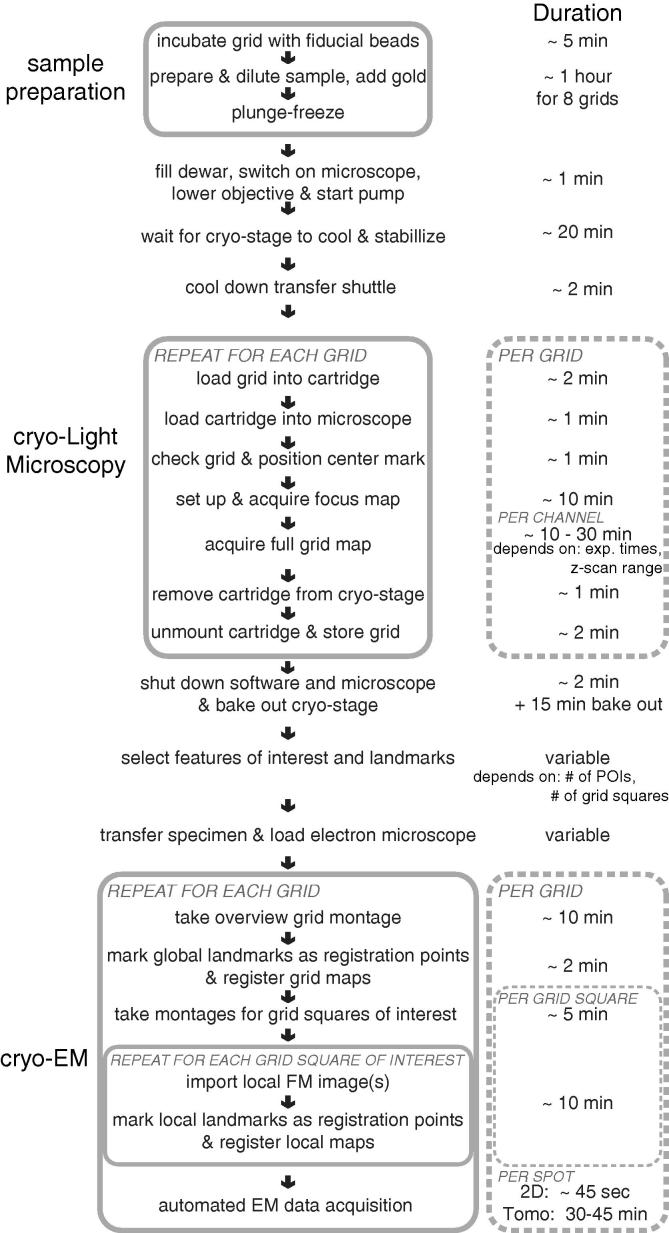
The cryo-CLEM workflow and estimated times for plunge-frozen specimens. The individual steps of the workflow are show in the left column, with estimated durations listed on the right. Procedures that should happen without a longer interruption or that need to be repeated are grouped in gray boxes.

Here we give an overview of the workflow for correlative cryo-FM and cryo-EM using the system. In the Appendix we present a detailed step-by-step protocol. A schematic illustration with approximate times is shown in Fig. 2.

#### 2.2.1 Sample and grid preparation

For cryo-EM imaging the sample is applied to metal (copper, gold) grids covered by a thin holey carbon support film. When choosing suitable grids for the cryo-CLEM experiment, the field of view (FOV) of the camera/microscope combination should be considered. It is convenient if an entire grid square fits within the FOV. In our case, with a FOV of 179 × 134 μm, we choose either 200 or 300-square mesh grids. We also observed that some types of support film, such as Quantifoil (Quantifoil Micro Tools GmbH, Groβlöbichau, Germany), exhibit auto-fluorescence particularly in the green channels when cooled, while in others (C-Flat, Protochip Inc., Morrisville, NC, USA) the effect is not as strong. This also needs to be considered when choosing the appropriate grid type.

Aligning and registering images between FM and EM is done in three steps. First, a rough registration is preformed using grid-wide landmarks that are visible in both imaging modalities. Irregularities in individual grid squares such as defects in support film or arrangements of sections/cells are typically sufficient landmarks. Alternatively, patterns of regular landmarks are available on commercial “finder-grids”. Second, we refine the coordinate registration between FM and EM locally to acquire high-resolution EM images or tomograms precisely at the desired positions. To maximize the correlation accuracy we perform a third, post-imaging registration step that makes use of plastic microspheres that are fluorescent in multiple channels and whose electron density is sufficient to identify them in EM images (Kukulski et al., 2011, Schorb and Briggs, 2014). We apply these fluorescent beads (TetraSpeck, Thermo Fisher Scientific, Waltham, MA, USA) to the support film of the grids before plunging as described in (Schorb and Briggs, 2014). The choice of beads is influenced by the fluorescence properties of the sample. The size and thus the brightness of the TetraSpecks should approximately match the expected intensity of the target signal in the desired channel. We typically use beads with a diameter of 100 nm while for very dim specimens 50 nm TetraSpecks that are 8 times less bright can be used. We also add 10 nm colloidal gold beads as alignment markers for cryo-ET or for the precise registration of images acquired at different EM magnifications (Kukulski et al., 2011). We vitrify the specimen by plunge freezing. The exact protocol for preparation of the EM grid will necessarily depend upon the sample the user wishes to study.

#### 2.2.2 Transfer of sample into cryo-FM stage

The cryo-FM transfer shuttle is cooled by liquid nitrogen and all transfer steps are performed in a cold, dry nitrogen gas atmosphere. The grids are transferred into the shuttle in standard, round, cryo-EM grid boxes. A single grid is loaded into the imaging cartridge. The imaging cartridge has two thin clips that secure the grids. These clips are released for loading and unloading of the grid using the cartridge loading station (CLS) (Fig.1D and Movie S1). To move the cartridge from the CLS into the microscope stage, it is picked up with the loading rod. The transfer shuttle as a whole is moved to the cryo-light microscope and docked to the entry port on the side of the cryo-stage. After opening the two shutters that seal the port, the loading rod is inserted into the cryo-stage. Once the cartridge is released in place, the loading rod is retracted, the shutters are closed and the transfer shuttle is removed.

#### 2.2.3 Cryo-light microscopy

To screen the overall condition of the specimen regarding ice quality and thickness, we perform a brief inspection by brightfield imaging. In order to register the grid with the coordinate system of the microscope we identify the grid’s center mark and store it in the software. As an EM grid is never perfectly flat, the next step is to generate a “focus map” of the grid to determine its topography. To speed up the procedure in the absence of bright fluorescence signals, a first coarse scan using brightfield illumination can be performed across the grid. Subsequently, the focus map is generated by autofocus scans using the fluorescence signal from the fiducial beads or the specimen.

The entire process from loading the grid into the cartridge until the grid-wide focus map is generated takes about 15 min per grid (Fig. 2). During data collection, we need to collect multiple images at different focal levels at each position (a “*Z*-stack”) to compensate for differences in sample height across the FOV. We therefore define the number of focal levels to be collected depending on the thickness and flatness of the sample (a typical *Z*-stack size is 10 μm with 1 μm increment). We also define the number of colour channels and adjust the illumination intensity and camera exposure time for each colour channel. The intensity settings usually stay the same for different grids from one batch while the settings for the *Z*-stack may differ from grid to grid.

Based on these settings, the CLEM acquisition software can run an automated scan of the entire grid, acquiring a multi-colour, multi-Z image stack at each *XY*-position of the grid. It uses image overlap to stitch the single tiles into a map of the full specimen. The time required to scan the grid depends on the number of desired channels as well as their exposure time and the *Z*-stack size. Typically a full acquisition takes about 10–30 min per fluorescence channel. Once the acquisition is complete, the cartridge is removed from the cryo-FM and the grid retrieved via the transfer shuttle and stored. The image data can be transferred to another computer for analysis while other grids are imaged.

**Fig. 3:**
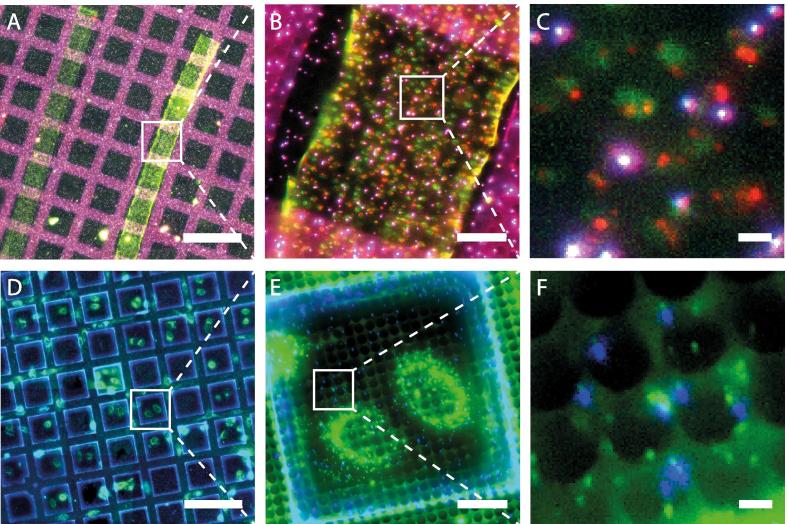
Cryo-FM imaging of frozen-hydrated sections and whole vitrified cells. **A** Stitched cryo-fluorescence mosaic image, as generated during the automated grid scan, of vitreous sections of high-pressure frozen yeast cells with alpha-subunit of COPI fused to mCherry. Two section ribbons of 60 nm (left) and 100 nm (right) nominal thickness are shown. Scale bar: 200 μm. **B** Zoom in on the grid square marked in A. Scale bar: 20 μm. **C** High magnification view of the box marked in B. The cytoplasm is slightly autofluorescent in the green channel, mCherry signal appears in red, TetraSpeck beads appear white/magenta and are fluorescent in green, red and far red (channel displayed in blue). Scale bar: 2 μm. **D** Cryo-fluorescence mosaic of a grid with plunge-frozen HeLa cells expressing COPII coat component Sec23 labeled with EYFP (Verissimo et al., 2015) grown on a gold EM grid. Scale bar: 200 μm. **E** Zoom in on the grid square marked in D. Scale bar: 20 μm. **F** High Magnification view of the box marked in E with EYFP signal displayed in green and TetraSpeck beads in blue (far red channel). Scale bar: 2 μm. All images shown are maximum projections of Z-stacks.

**Fig. 4:**
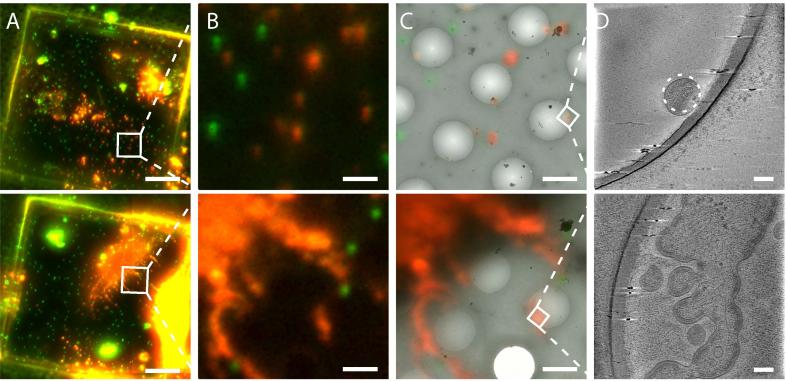
Correlated cryo-FM and cryo-ET. HeLa cells were transfected with the HIV-1-derived plasmids pCHIV and pCHIV.mCherry (Lampe et al., 2007), as described in (Baumgärtel et al., 2011) and vitrified by plunge-freezing. **A** Grid square overviews. TetraSpeck beads appear in green (imaged in far red) and HIV Gag-mCherry signal in red. Scale bar: 20 μm. **B** Zoom into the marked areas of interest. Scale bar: 2 μm. **C** Superposition of the cryo-EM montage of the selected area and the transformed fluorescence image after correlation. Scale bar: 2 μm. **D** Central section through a tomogram collected at the marked point of interest. Upper panel shows an individual mature HIV-1 particle. The dashed circle marks the predicted position of the fluorescent signal as determined by post-acquisition high-accuracy correlation. Lower panel displays virus assembly sites at the plasma membrane of the cell. Tomograms were collected on a Titan Krios electron microscope (FEI) equipped with Quantum 967 LS energy filter and K2 direct detector (Gatan) at a pixel size of 1.32 Å and reconstructed using IMOD (Kremer et al., 1996). Scale bar: 50 nm.

**Fig. 5:**
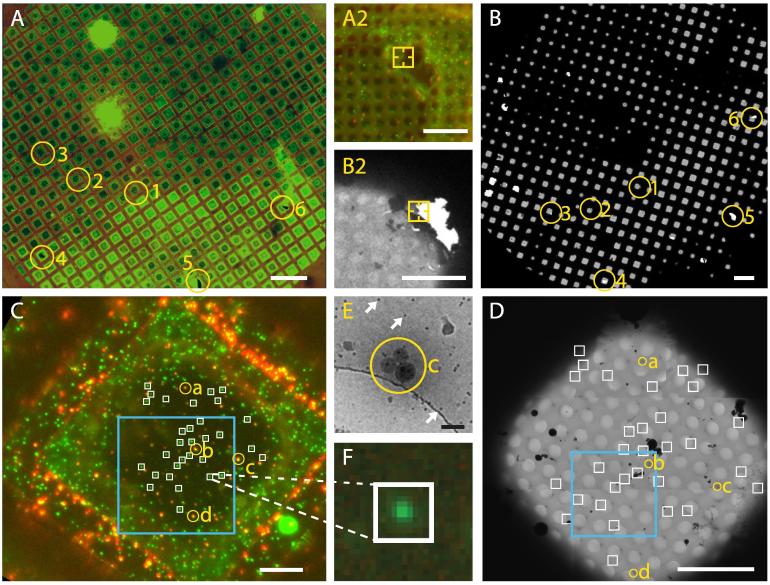
The procedure to set up automated EM acquisition at fluorescent POIs. **A** Cryo-fluorescence mosaic of the whole grid, merge of red and green channels. The yellow circles indicate grid-wide reference landmarks such as torn support film. Scale bar: 200 μm. The inset **A2** is a magnified view within circle 2 showing a typical grid-wide landmark: a corner of a hole in the support film. Scale bar: 10 μm. **B** Full cryo-EM grid map acquired by SerialEM. The same landmarks as in A are marked. The inset **B2** shows the same feature as marked in A2. Scale bar: 10 μm. **C** Magnified FM view of a single grid square. Virus particles appear as green spots while the multi-colour fluorescent beads (TetraSpecks) are visible in both channels. Yellow circles indicate landmarks used to locally align this image with the corresponding EM acquisition. As local landmarks we typically use pairs or clusters of fiducial beads, which appear as bright fluorescent signals. White boxes indicate fluorescent signals marked as target POIs to be automatically acquired in EM. The blue rectangle indicates the part of the image used for the post-acquisition high-accuracy correlation illustrated in Fig. 6A. Scale bar: 200 μm. **D** Cryo-EM mosaic acquisition of the same grid square. Yellow circles indicate local landmarks used to locally register the FM image of the grid square (C). White boxes indicate the positions where high-magnification images were recorded based on the transformed positions of the fluorescent POIs. The blue rectangle indicates the region in the grid square where the post-acquisition high-accuracy correlation illustrated in Fig. 6B was performed. Scale bar: 10 μm. **E** Magnified view of the landmark feature c, which is a cluster of three ∼100 nm TetraSpeck beads. White arrows indicate the 10 nm gold fiducials. Scale bar: 200 nm. **F** Magnified view of the cryo-FM image showing a fluorescence signal of interest. The white box corresponds to the FOV of 1.24 μm of the high-magnification EM image automatically acquired at this spot barely covering 10 × 10 FM pixels.

**Fig. 6:**
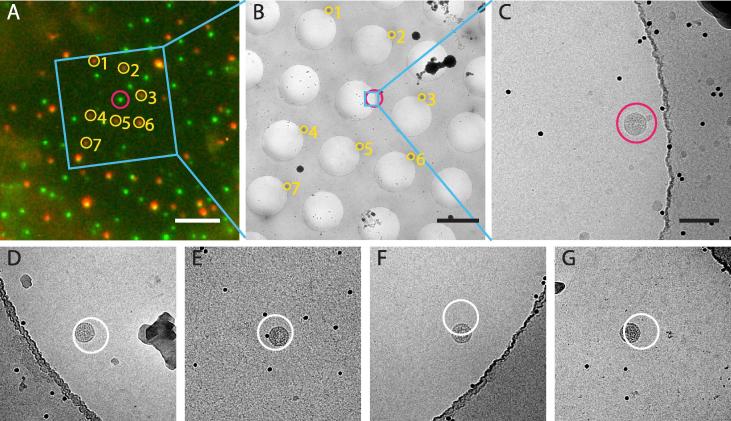
Post-acquisition high-accuracy correlation. **A** The area of interest in the FM image (marked with blue box in Fig.5C) surrounding the signal of interest circled in red. The TetraSpeck beads that are used for the high-accuracy correlation are marked with yellow circles. The blue area indicates the location of the image shown in panel B. Scale bar: 5 μm. **B** Single medium-magnification cryo-EM image of the region corresponding to the blue boxes in Figs. 5C, 5D and 6A, acquired after the high-magnification imaging at the POI is finished. The positions of the TetraSpeck beads corresponding to those in A are marked in yellow. The blue square indicates the position and FOV of the high-magnification image in C. The red circle is centered on the predicted coordinates of the feature of interest calculated by the post-acquisition correlation procedure using the fiducial beads. Scale bar: 2 μm. **C** High-magnification image at the POI. The red circle marks the coordinates predicted by post-acquisition high-accuracy correlation. The circle has a radius of 50 nm. Scale bar: 100 nm. **D** to **G** images and analogous coordinate predictions for four other particles. Image E was acquired on the carbon support film. Radius of the white prediction circles: 50 nm. FOV: 500 nm.

The Cryo-FM system is compatible with different cameras. Images in this manuscript were collected on a Leica DFC-365FX (Figs. 3A–C, 5, 6) or a Hamamatsu ORCA-Flash4.0 (Figs. 3D–F, 4).

#### 2.2.4 Annotation of FM images to define positions of interest

The cryo-FM images are next analysed to determine the positions of interest (POI) where EM acquisition should take place. This can be done by manual selection of fluorescent signals (as was done in this study), or combined with image analysis routines (e.g. particle picking) within the FM control software (LAS X) or using another software. We also mark the coordinates of grid-wide landmarks, such as broken grid squares or marks on grid bars. These are called “Grid reference positions” within the software. The two coordinate lists are saved in a file format that can be directly read by the software package used for EM.

#### 2.2.5 Cryo-electron microscopy

The grids are then loaded into the EM. We control the EM using the software package SerialEM (Mastronarde, 2005). It is capable of mapping imported images and associated coordinate systems onto the EM’s internal stage coordinates (Briegel et al., 2010, Mastronarde, 2016). Its “navigator files” can store the coordinates of selected points as well as links to image files and associations to other coordinate systems. The coordinate lists and cryo-FM images can be opened and visualised inside SerialEM.

We collect an initial overview montage of the entire grid using SerialEM. Within the EM montage we then mark the grid-wide landmarks previously selected in the FM images. Using the matching pairs of landmarks as registration points, SerialEM then transforms the grid-wide FM coordinates onto the EM coordinate frame. The accuracy of the grid-wide coordinate registration is sufficient to target grid-squares containing POIs. We next record montaged EM maps at an intermediate magnification covering each grid square of interest and perform a second, local, coordinate registration from FM to EM on the grid square level using the fluorescent beads as fiducial markers. The POIs previously identified by FM have now been located in the EM coordinate frame. High-magnification cryo-EM images or tomograms can then be collected at all POIs in an automated manner.

#### 2.2.6 Post-acquisition high-accuracy correlation

After the acquisition, the FM and EM images can be more accurately aligned using the TetraSpeck beads as fiducial markers (Fig. 6). Based on coordinates of these fiducial markers, the mathematical transformation that relates the FM and EM images can be calculated. Identification of the beads in the FM and EM images and alignment of the images can be directly performed using the scripts described in (Kukulski et al., 2011) which are implemented in MATLAB (MathWorks, Natick, MA, USA). These scripts, with modifications that adapt for file formats and further improve ease of use, were used for the experiments described here and are available for download from http://www.embl.de/download/briggs/cryoCLEM/index.htm. With this post-acquisition bead-based registration we can achieve high correlation accuracy of ∼100 nm or less. Other software packages such as Icy (de Chaumont et al., 2012, Heiligenstein et al., 2016) can also be used.

### 2.3 Experimental performance

The design of the stage and particularly the transfer shuttle has reduced contamination of the sample by atmospheric humidity during transfer as compared to our previous instrument (Schorb and Briggs, 2014). Inside the cryo-stage, contamination is negligible and multiple grids can be imaged over 6 h without the need of a bake out. The cryo-FM stage provides mechanically stable imaging conditions. During the typical imaging period for one grid (about one hour for acquisition of a full grid map in three channels) mechanical drift is minimal, and is not sufficient to affect the focussing of tiles or the alignment of the full mosaic. For the duration of a single exposure we do not observe vibrations of the stage that would blur the image.

Together with the associated software that allows automated cryo-FM imaging, the new system provides an efficient cryo-CLEM workflow. The key advantages of this workflow are the ability to run automated mosaic cryo-FM scans covering the complete grid, and the ability to subsequently run automated cryo-EM data collection on the selected POIs. The entire process, from sample preparation and cryo-FM imaging to targeted acquisition of 300 high-magnification cryo-EM images or 25 tomograms can be performed within 2 days. Freezing and FM of 6 grids can be achieved in one standard working day. Signals of interest are then identified and a second day is spent on loading the EM, mapping grids, aligning the coordinate systems, setting up the imaging conditions and running the automated acquisition which can continue overnight.

### 2.4 Proof of principle applications

Representative cryo-FM grid-scans of plunge-frozen cells and vitreous sections are shown in Fig. 3. An example of the use of the system to perform targeted cryo-ET is shown in Fig. 4. In this example, cells transfected with a Human immunodeficiency virus (HIV-1) variant where the Gag protein is tagged with mCherry (Lampe et al., 2007), were vitrified by plunge freezing and imaged by cryo-FM. Target positions were identified based on the presence of red signals in the cryo-FM image that were located within regions of appropriate ice thickness in montaged intermediate magnification cryo-EM maps. These positions were imaged by cryo-ET revealing virus assembly sites and recently released virus particles.

To demonstrate the use of the system for automated data collection, we applied the full workflow to plunge-frozen, fluorescent p22 bacteriophage particles as previously described in (O’Neil et al., 2012, O’Neil et al., 2011; Schorb and Briggs, 2014). These provide fluorescent point sources that can be unambiguously identified in cryo-EM, and therefore allow quantitative assessment of the correlation performance. We generated full mosaic scans of two grids by cryo-FM (Fig.5A, acquisition parameters and detailed protocol, see Appendix). Based on the FM images we identified 9 grid squares of interest, distributed over the two grids, and marked 423 fluorescent signals as POIs (Fig.5C). We then collected high-magnification images of the POIs. For this experiment we used an FEI Polara microscope (FEI Company, Hillsboro, OR, USA) equipped with GATAN US4000 CCD (Gatan Inc., Pleasanton, CA, USA) camera operated at 100 kV at 31000x magnification with 3.5 μm underfocus and an electron dose of 20 e/Å^2^ per image. The whole experiment from freezing to data collection was performed in 2.5 days.

To verify the success of the correlation procedure, we analysed a subset of 100 acquired images. In these we found 87 virus particles within the FOV (1.24 μm squared). In 5 images there was aggregated material that is likely to have given rise to the fluorescence signal while 1 image contained a fluorescent bead instead of a virus. In 5 images we found nothing, and for 2 images the EM acquisition failed. For 98 successful image acquisitions, 93 contained the fluorescence signal source, a hit-rate of 95%. To assess the possibility of imaging a fluorescent specimen by pure chance, we set up 20 acquisitions randomly positioned in a neighbouring grid square. In these we found 1 virus and 1 fiducial bead within the FOV while 18 images contain no visible feature (10% hit-rate). We next used the deviation of the positions of the fluorescent objects from the center of the images to estimate the accuracy of image acquisition. This deviation is approximately normally distributed with a standard deviation of 227/256 nm (in *XY* direction), which is consistent with the observed 95% hit-rate. This distribution indicates that data could be acquired with a FOV of 900 nm while still expecting an 80% hit rate. The largest source of inaccuracy during this step is imperfect registration of images during image montaging within the SerialEM software. We have been able to obtain improved targeting precision when using other software such as IMOD to generate image montages (Kremer et al., 1996).

We then performed post-acquisition high-accuracy coordinate registration on a subset of the positions exactly as described in (Kukulski et al., 2011, Schorb and Briggs, 2014). Most users take about 5 min per registration for this step. For 52 high-magnification images, where the predicted location of the fluorescence signal is clearly associated with a virus-like particle visible in the image, we measured the deviation of the predicted coordinate from the center of the observed particle (Fig. 6). This deviation is normally distributed for each coordinate axis with standard deviations of 31 and 37 nm in *X* and *Y*. We note that the obtained accuracy for a specific sample will be dependent on multiple factors, including the intensity of the fluorescent signal (and therefore the ability to define its center) and the distribution, density and intensity of the Tetraspecks.

## 3 Discussion

Here we have introduced new hardware and software for correlative cryo-FM and cryo-EM and described a workflow by which they can be used for semi-automated imaging. The stage and transfer shuttle permit use of a 0.9 NA short working-distance objective. The system offers high stability and reduced contamination to facilitate imaging. The instrument together with the dedicated software provides the capability of doing automated scans of full cryo-EM grids. Together these developments have provided us with a dramatic increase in throughput and reliability when compared to our previous system (Schorb and Briggs, 2014). The workflow enables an offline selection of coordinate lists for both landmarks and POI before going to the EM. These lists can then be directly imported to the EM control software and used as basis for automated acquisition.

The cryo-CLEM system can be used to study specimens where there is only a single target signal per grid or numerous events separated only by the diffraction limit of the fluorescence microscope. Where the fluorescent signal can be identified above background it should be possible to locate it in the electron microscope. We envisage that the ease-of-use of the workflow makes it feasible to add cryo-FM as a routine step between sample preparation and cryo-EM/ET data collection. In particular for cellular and other heterogeneous samples this will facilitate efficient and targeted cryo-EM data collection, while providing complimentary information.

## Conflict of interest statement

M.S., L.G., R.L. and J.A.G.B have filed patents related to this work. Intellectual property therein has been licensed by Leica Microsystems who have commercialized the cryo-FM system and released it as a product.

## Figures and Tables

**Supplementary Fig. S1:**
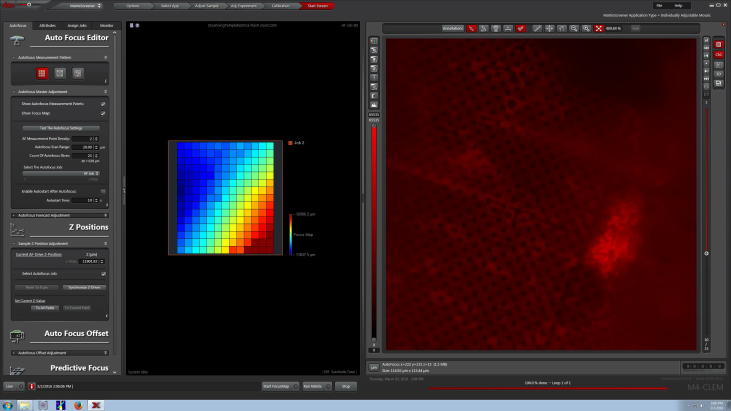
Screenshot of the MatrixScreener CLEM module. Start Screen tab, Autofocus panel. The right half of the screen shows the current image (same cells as in Fig. 3D–F). The center shows the measured and interpolated focus map with the parameters to set up the measurement on the left.

**Supplementary Fig. S2:**
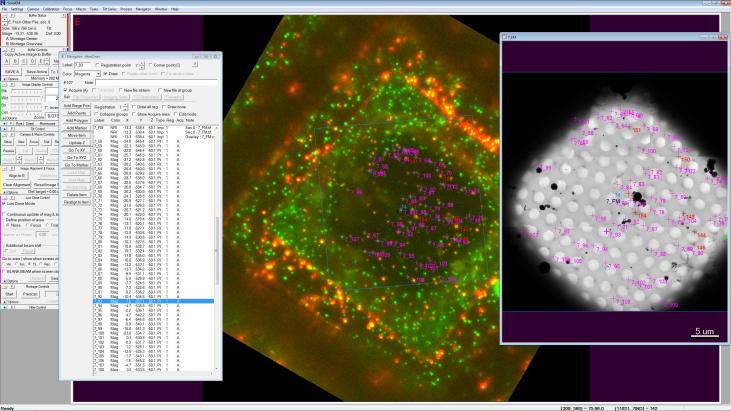
Screenshot: Setting up automated EM acquisition in SerialEM. The navigator window showing the coordinate list of all points and map images is seen in the foreground. The POI selected for acquisition are shown as pink crosses on top of both FM and EM maps. These have been aligned using the registration points shown in red. The positioning of the EM map on the right is indicated as the overlaid blue box on top of the FM map.

## Appendix A Appendix – Detailed protocol

### Software layout

The CLEM module developed for Leica’s LAS X microscope control software is based on the MatrixScreener HCSA module. It allows the acquisition of tiled mosaic image stacks of any rectangular shape. The software displays the last acquired image or live view on the right half of the screen and a set of tabs at the top of the left half (Fig. S1). These tabs serve to set up individual parts of the workflow.

*Options –* sets the general options for the MatrixScreener HCSA environment like output formats, directory structures etc. and does not need to be modified for each experiment.

*Select App –* allows loading predefined experiments (*Matrix Applications*). These presets contain all information on the geometry and imaging parameters. You could set up an individual *Matrix Application* for each type of specimen you want to acquire, or for the different purposes such as acquiring a whole plunge-frozen grid versus only a subregion that contains CEMOVIS sections.

*Adjust Sample –* is not relevant for the CLEM workflow.

*Adjust Experiment –* this is where all imaging parameters are set. The tab is visually almost identical to the LAS X main window but allows you to define the parameters for individual jobs (listed at the top under *collecting pattern*) instead of for a single imaging run. Here we will define settings for the imaging job plus one or more jobs for autofocusing.

*Calibration* – this tab defines the geometry of the acquisition area and the tiles where the imaging job should be run. You can also select a subregion to test the acquisitions (the *Calibration* run).

*Start Screen* – here the global acquisition is set up. With the *Autofocus* tab on the left the focus map is generated, some options during runtime are set in *Attributes* and the scan is initiated by clicking *Run Matrix* on the bottom right.

### Cryo-FM preparation

– Switch on the microscope, microscope smart touch panel (STP), pump controller, PC and other microscope components such as camera and light source.
– With the STP, move the objective to the focus position to ensure proper cooling of its front. The STP remembers the stored focus position from the last experiment.
– Fill the dewar with LN, insert the pump and press the cool button to start cooling the cryo-stage to -195°C (cooling takes about 20 min).

### Loading a grid into the cryo-FM stage

– Once the cryo-stage has cooled to the target temperature, fill the transfer station with LN to cool it down. The LN level should be below the port that connects the transfer system to the microscope (Fig.1B). Wait until bubbling stops at all metal surfaces to ensure all parts are completely cold. At this stage double check the LN levels in the main dewar and refill if necessary.
– Load a grid into the cartridge (Fig.1D, movie S1): Place a cartridge into the cartridge loading station (CLS). Open the cartridge clips by moving the pin in the CLS (using the dedicated tool) and raising it (note that the direction in which the pin should be moved depends on the orientation in which the user chooses to insert the cartridge into the CLS. This will depend on whether the user prefers to work with right or left hand). Slide the grid into place and close the clips by pushing the pin down and moving it back to the central position. Take care to initially push the pin exactly vertically, as otherwise it will not move out of the resting positions easily.
– Grab the cartridge with the insertion rod’s gripper and retract it. Move the CLS out of the rod’s path.
– Attach the transfer shuttle to the cryo-FM stage (Fig.1E) and, using the STP control, raise the objective by 2 mm.
– Open the locks between transfer shuttle and stage (one slider on the stage itself and one on the transfer shuttle). Load the cartridge into the cryo-FM stage by gently pushing the insertion rod into the stage and releasing the cartridge by opening the gripper (Fig.1F). Retract the insertion rod, close locks and remove the transfer station. Lower the objective back to its focus position.

### Preparing the software

Set up the software and initialize a CLEM experiment as follows:

– Launch LAS X (standard widefield configuration) software and switch to the MatrixScreener CLEM module using the drop-down selector at the top left of the window.
– In the *Applications* tab select the preset application that matches the desired experiment, in this example we use a full cryo-CLEM grid scan. Be aware that upon initialization of the preset the stage will move to the position defined by the preset.
– The *Experiment* tab should now show the different jobs for autofocusing and imaging. These settings are loaded with the experiment preset.

### Setting up imaging conditions

The *Adjust Experiment* tab serves to set up the imaging conditions for the jobs listed in the top of its main window. Jobs can be defined for image acquisition or the autofocus (AF) routine and can be composed of multiple channels that are listed below. For each of the channels the panels on the left can be used to adjust the imaging parameters including: the choice of filter cubes, illumination settings, camera exposure times or whether a single image or an image stack over different z values is desired. The image panel on the right side of the window, can be used to define a region of interest to restrict the imaging area.

– Set up appropriate imaging conditions for both AF and imaging acquisition. The choice of conditions depend upon the sample being imaged. We recommend that the field of view (FOV) is limited to the center of the camera while running an AF job, and that camera binning is applied. This ensures that the focus value measured is that for the center of the area of interest, and reduces imaging time.

### Setting up the acquisition area

When a tile scan of a whole grid is desired, as it typically is the case for plunge-frozen samples, an appropriate acquisition area is a square area of about 2 × 2 mm. For the field of view of the objective and camera used here for the automated data collection (Leica DFC-365FX), with an ideal image overlap of 18%, this requires 13 × 15 image tiles. When using the system with a Hamamatsu ORCA-Flash4.0, we use 9 × 9 tiles. The acquisition area settings only need to be set up initially and are then stored in the CLEM application for all future experiments. Then the software’s tile positioning is aligned with the physical location of the grid inside the cryo-stage as follows:

– Go to the *Calibration* tab and start the illumination by clicking *live.* We usually do this alignment using brightfield illumination. Click the central tile in the software to move to the currently stored position.
– Move the stage to the true center of the grid using the STP control and update the central tile position by clicking *Adjust Mosaic position* (*Calibration* tab, *Mosaic Editor*). Now the software tiles and the grid are aligned.

As an alternative, when only a subregion of the grid needs to be scanned (for example when imaging CEMOVIS sections, the geometry can also be defined by providing the software with the coordinates of the top-left and bottom-right corner of the desired scan area in the *Calibration* tab.

### Generating the focus map

The software finds the focus by acquiring a set of images at different Z positions and then determining the slice with maximum contrast. In order to have a proper focus position for all acquisition tiles during data collection, the software requires a topographic map of the specimen with a focus position assigned to each tile. To generate this topographic “focus map”, the software performs AF measurements at a subset of tiles and interpolates the values for the remaining tiles. The focus map is displayed as a heat map overlay on top of the tile grid (Fig. S1).

Depending on the sample, the user can adjust the density of tiles where focus will be measured (every n tiles, where n is the *Measurement point density*), the range over which focus will be scanned in μm (*Scan range*) and the number of images to be taken within that range (*Count of slices*). When imaging an entire grid, we found that the best compromise between precise local focus measurements and the time required to generate the focus map is achieved by performing an initial rough focus mapping, either in brightfield or using a fluorescence channel featuring very bright signals, followed by a finer focus mapping using one of the fluorescence channels. When scanning a smaller region of the grid, or if there is a particularly bright fluorescence signal present in the specimen, the AF can be entirely done using fluorescence channels. In the protocol below we start with a coarse brightfield AF job and then use a far-red fluorescence signal e.g. from TetraSpeck fiducial beads for finer focus mapping.

– Go to the *Start Screen* → *Autofocus* tab (Fig. S1).
– Set parameters for coarse mapping using either the brightfield or a bright fluorescence AF job. We typically use these parameters: *Measurement point density* 7, *Scan range* 50–75 μm, *Count of slices* 15–21.
– Start the scan by clicking *Start FocusMap.*

If the autofocus fails to identify the focus position in a particular tile, the focus can be manually adjusted (click the tile during *live* imaging → focus manually (STP) → click *set to current field* to store the current Z position in the focus map).

The focus values for the tiles in between the measured fields are automatically interpolated and the focus map is updated. The values stored in the focus map will be used as starting values for subsequent refinement scans. If you interrupt a focus scan its values are discarded.

– Refine the focus map by running a second scan with a higher focus point density and a more precise step size. This time choose the fluorescence autofocus channel for the measurement (typical settings: *Measurement point density* 2, *Scan range* 10–15 μm, *Count of slices* 11–17). You now have an accurate focus map of the entire grid based on the signal of the fiducial beads on the carbon support film or on bright fluorescent features within your specimen.
– For very long scans with many fluorescence channels or long exposure times, you can enable drift compensation for a subset of fields (in *Start Screen* → *Attributes*). This means that when these tile positions are reached during the acquisition scan, a AF job is run and the global focus map is adjusted accordingly. The active tiles are shown with pink borders.

### Setting up the full grid acquisition

– Gradients in the sample mean that there may be no focus position at which the entire camera FOV is in focus. For this reason imaging at multiple focus positions is recommended. This can be achieved by setting a multi Z-slice acquisition (a Z-stack). Typical parameters are: 10 μm thickness, 1 μm increment.
– You can correct for differences in focus between channels by activating and setting up relative focus correction (*RFC*).
– The acquisition and imaging settings can be tested during a local calibration run on few neighbouring tiles. To start, go to the *Calibration* tab. Select a small subset of mosaic tiles to generate the test data and use *right-click* → *Mosaic calibration fields – enable selected scan fields*. Then start the calibration by clicking *Start Calibration*. The microscope will now acquire the selected fields with the given settings and stitch the resulting images in a montage for the user to assess.
– If you are happy with the result, start acquiring the full grid-wide mosaic by clicking *Start screen* → *Run Matrix*. The software will offer to store the experiment settings updating the presets in the currently active *MatrixScreener Application*.

### Removing the sample from the cryo-FM stage

– Following the successful completion of data acquisition, attach the cooled transfer shuttle to the cryo-stage (Fig.1E). Raise the objective by 2 mm, open the locks, insert the insertion rod and pick up the cartridge.
– Retract the rod, close the locks, remove the transfer shuttle and lower the objective back to the focus position.
– Move the cartridge from the insertion rod into the cartridge loading station (Fig.1D). Retract the rod. Retrieve the grid from the cartridge and store it.

### System shutdown

– Move the objective to its top position to protect it during heating, then push *heat* on the pump controller to start stage bake out. Wait 10 min to let the pump hose warm up and become flexible.
– Remove the pump from the dewar and store it in upright position to dry. Switch off the pump controller, microscope controllers, light source, etc. and empty the dewar to let it dry.

### Select features of interest and landmarks

The identification of fluorescent signals of interest can be performed in various software packages. We typically use two approaches to create coordinate lists that we can transfer to SerialEM and store as “navigator files”. One option is to load the FM images directly into SerialEM (*Navigator* → *Import Map*) and use the feature *add points* in SerialEM’s navigator window to mark the coordinates of FM. This does not have to be done at the computer controlling the EM as it is possible to install the SerialEM software on any standalone PC running Microsoft Windows. The navigator file can then be saved and transferred to the PC controlling the electron microscope. This approach might require manual editing of the navigator file in a text editor to modify its file paths linking to the images. An alternative option is to use the CLEM ClickTool which we have incorporated into the Leica cryo-CLEM software as follows:

– load your dataset by clicking the cloud symbol and selecting the source directory of the mosaic scan. The software displays individual slices and channels from the acquired image stack. You can navigate through these single mosaic slices with the slider on the bottom right.
– Identify *Grid Reference Positions*, global landmarks that will be used to register the mosaic scan with the full grid scan later collected in the EM. These can be marks on a finder-grid, broken grid squares, or other large features. Activate their selection (crosshair with red box) and click each landmark in the mosaic.
– You can copy and paste already clicked points from one image slice to another by clicking the *C* and *P* underneath the overview panel box.
– Continue the clicking with features that you identify in the remaining slices. It is important to first paste the existing coordinates from other slices to avoid confusion in the numbering.
– You can then mark the fluorescent signals for which you want to collect EM images. These are selected as *image positions of interest* (POI) in the same way.
– Save the coordinates for re-opening them in the ClickTool and/or export them as navigator files for further use in SerialEM.
– Concatenate the two navigator files for global reference positions and POI. This can be done in SerialEM by clicking *Navigator* → *Merge File* (or by using a text editor and saving the concatenated file).

Fluorescent signals of interest can also be identified using peak picking algorithms or other automated approaches. Some functionality to do this is directly available in LAS X. Other image analysis software could be used, in which case the user would have to format the output coordinates as a SerialEM navigator file.

For samples that have not been thinned by CEMOVIS or FIB milling, once the user is familiar with the sample the brightfield and fluorescent images can often provide a rough guide to local sample thickness. Signals of interest should only be marked in regions that are expected to be thin enough for cryo-EM imaging.

### Cryo-TEM acquisition

– Load the grid into the EM under cryo conditions using the appropriate cartridge or holder.
– Start SerialEM with the proper calibrations and settings for the desired acquisition voltage.
– Load the navigator file into SerialEM (*Navigator* → *Read & Open*)
– Set up a full grid montage (*Navigator* → *Montaging & Grids* → *Setup Full Montage*) at low magnification (110×, take care to remove the objective aperture) and start the acquisition.
– Once the mosaic is acquired, confirm the dialog to make it a map. In order to display the acquired mosaic image at full quality set the overview binning settings to the minimum and reload the map by double-clicking its entry in the navigator list.
– Load the fluorescence map by double-clicking the respective navigator entry. The image containing all associated points appears in the main window. (Fig. S2) Additional images can be imported using *Navigator* → *Import Map*. SerialEM offers to generate colour overlays of multiple channel FM images in the import dialogue (*Make colour overlay*).
– Open EM and FM grid maps in separate windows. (*Window* → *New Window*).
– Identify and click the features in the EM map that correspond to the *Grid reference positions* previously selected in the FM image using *Add Points* in the navigator window.
– Make both sets of landmark points *Registration points* by ticking the box in the navigator window. Assign each point to its partner from the fluorescence map and registration (1R1 with 2R1 etc.).
– Save the navigator file.
– Register the two coordinate systems by clicking *Navigator* → *Transform Items*. The target registration set in the navigator window should correspond to your EM grid map. Now all the points from the fluorescence images should be mapped on top of the EM grid map. This is sufficient to assign the grid squares of interest for further intermediate-magnification acquisition.
– Set up the *low dose* imaging states now, because SerialEM will remember the imaging settings used for collection of maps in the next step and will link them to all future acquisitions based on these maps.
– Acquire mosaic maps at an intermediate magnification for each grid square of interest. Select a magnification that maximizes the field of view while still ensuring that features to be used for local registration (for example TetraSpeck fiducial beads) are clearly visible (we use 3100×, or the lowest available SA magnification, bin 1, 10% or minimum 1 μm overlap). Start the acquisition of the maps using *Navigator* → *Acquire at points*.
– The global registration is not sufficiently accurate to acquire high-magnification images and locate the target feature in the correspondingly small FOV. Therefore we need to perform a refined registration for each grid square of interest individually using local landmarks (individual TetraSpecks, clusters of beads, other added fiducials or imperfections visible in both FM and EM). The procedure is the same as that described for the global maps. The pair-wise assignment of registration landmarks needs to be unique for each grid square, which may be achieved by re-indexing the EM registration points in the navigator file. If the POIs have been previously identified, for example using the ClickTool, then also reassign the POIs within the grid square to the respective registration. To do this, each point can be edited individually within SerialEM’s navigator window, or a text editor can be used to edit the registration entries in the navigator file.
– When registered, the target coordinates from FM are accurately shown in the EM maps (Fig. S2). We can then set up the automated EM acquisition in SerialEM by enabling the POIs for acquisition in the navigator window.
– Acquire high-mag 2D images or tomograms at the desired magnification at all positions of interest using *Navigator* → *Acquire at points*. We have set up an acquisition macro that contains the command *RealigntoNavItem* to accurately match the exact position of acquisition to the feature environment within the lower magnification map image. At each of the positions we also acquire a low-magnification overview image after high magnification data acquisition using the acquisition macro. We will later use this single image for the high-accuracy correlation using the fluorescent beads and thus avoid inaccuracies caused by the stitching of mosaics.

## Appendix B Supplementary data

Supplementary Movie S1 This movie demonstrates the workflow for: preparation and cooling of the stage and transfer shuttle, inserting a grid into the cartridge, and loading of the cartridge into the cryo-stage.
