# Supplementary figures and images for: New hardware and workflows for semi-automated correlative cryo-fluorescence and cryo-electron microscopy/tomography
Source: J Struct Biol. 2017 Feb;197(2):83–93. doi: 10.1016/j.jsb.2016.06.020 (PMC5287355; doi:10.1016/j.jsb.2016.06.020)

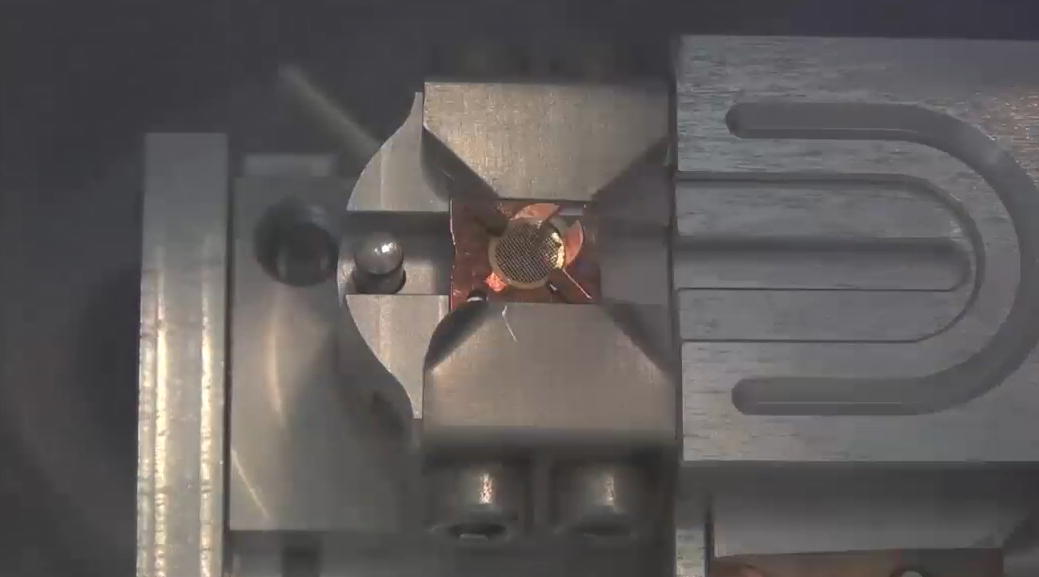

Supplement: Supplementary Movie S1 — This movie demonstrates the workflow for: preparation and cooling of the stage and transfer shuttle, inserting a grid into the cartridge, and loading of the cartridge into the cryo-stage. [file mmc1.jpg]
